# Synthesis and Biological Evaluation of Genistein-IR783 Conjugate: Cancer Cell Targeted Delivery in MCF-7 for Superior Anti-Cancer Therapy

**DOI:** 10.3390/molecules24224120

**Published:** 2019-11-14

**Authors:** Yang Guan, Yi Zhang, Juan Zou, Li-Ping Huang, Mahendra D. Chordia, Wei Yue, Jin-Jun Wu, Dong-Feng Pan

**Affiliations:** 1 College of Pharmacy, Jiangxi University of Traditional Chinese Medicine, Nanchang, Jiangxi 330004, China; guanyang1671@163.com (Y.G.); jxnchlp@163.com (L.-P.H.); 2 International Institute for Translational Chinese Medicine, Guangzhou University of Chinese Medicine, Guangzhou, Guangdong 510006, China; zoujuangzy@163.com; 3 Department of Radiology and Medical Imaging, Charlottesville, VA 22903, USA; zhangy87@gmail.com (Y.Z.); mdc3x@virginia.edu (M.D.C.); 4 Department of Endocrinology, University of Virginia, Charlottesville, VA 22903, USA; WY9C@hscmail.mcc.virginia.edu

**Keywords:** genistein, breast cancer, targeted drug delivery, tumor targeting, near infrared

## Abstract

The flavonoid-based natural product genistein is a biologically active compound possessing promising anti-oxidant and anti-cancer properties. Poor pharmacokinetics along with low potency limit however the therapeutic application of genistein in cancer therapy. In order to overcome those limitations and to expand its therapeutic window of efficacy, we sought to covalently attach genistein with a heptamethine cyanine dye—IR 783—for cancer cell targeting and enhanced delivery to tumors. Herein we report the synthesis, a selective detailed characterization and preliminary in vitro/in vivo biological evaluation of genistein-IR 783 conjugate **4**. The conjugate **4** displayed improved potency against human breast cancer MCF-7 cells (10.4 ± 1.0 μM) as compared with the parent genistein (24.8 ± 0.5 μM) or IR 783 (25.7 ± 0.7 μM) and exhibited selective high uptake in MCF-7 as against the normal mammary gland MCF-10A cells in various assays. In the cell viability assay, conjugate **4** exhibited over threefold lower potency against MCF-10A cells (32.1 ± 1.1 μM) suggesting that the anti-cancer profile of parent genistein is significantly improved upon conjugation with the dye IR783. Furthermore, the genistein-IR783 conjugate **4** was shown to be especially accumulated in MCF-7 cancer cells by fluorescent intensity measurements and inverted fluorescence microscopy in fixed cells as well as in live cells with time via live cell confocal fluorescence imaging. The mechanism-based uptake inhibition of conjugate **4** was observed with OATPs inhibitor BSP and in part with amiloride, as a macropinocytosis inhibitor. For the first time we have shown amiloride inhibited uptake of cyanine dye by about ~40%. Finally, genistein-IR 783 conjugate **4** was shown to be localized in MCF-7 tumor xenografts of mice breast cancer model via in vivo near infrared fluorescence (NIRF) imaging. In conclusion, conjugation of genistein with cyanine dye IR783 indeed improved its pharmacological profile by cancer cell selective uptake and targeting and therefore warrants further investigations as a new anti-cancer therapeutics derived from natural product genistein.

## 1. Introduction

Genistein, one of the oldest known isoflavone-based natural products, is isolated from a variety of plants, especially from soy and fava beans. It displays a wide range of biological activities, including general antioxidant and anticancer properties. Currently it is under extensive research investigations for anti-cancer activity against various types of tumors, especially breast cancer [1,2]. Numerous bio-molecular targets are established for the observed bioactivity of genistein in cancer and other cells. These include but are not limited to estrogen receptors (ER), protein tyrosine kinases (PTK) [3], mammalian DNA topoisomerase II, peroxisome proliferator-activated receptors (PPARs) etc. In some cases genistein is known to activate/stimulate a biological target, for example it acts as an agonist on ER [4] or activates PPARs [5,6], while in other cases it inhibits/suppresses some biological function of the target, for example DNA topoisomerase [7,8]. All in all genistein offers beneficial health effects to humans through these bioactivities. However, genistein exhibits paltry oral bioavailability and poor pharmacokinetics, which are unsuitable characteristics to develop it as an anticancer agent [9,10]. Selective delivery of genistein to cancer cells is necessary to improve its anticancer pharmacological profile considering the promising biological activities it possesses [9,10]. Genistein’s low toxicity at higher doses allows further structural modifications for selective cancer-cell targeting and delivery to extract higher therapeutic value. In this regard, to explore optimal potential of genistein as therapeutics a structural modification by conjugating it with cancer cell targeting molecules seems to be a highly attractive scientific endeavor.

Cancer-cell-specific targeting and tumor delivery of anticancer agents are considered a “Holy Grail” of drug development and one of the most widely sought but highly complex technologies. Many tools and techniques have been developed and applied to achieve this goal, for example macromolecular assemblies such as nanoparticles, nano-emulsions, micelles, liposomes and antibody conjugates [11,12,13]. Especially, in the case of genistein, liquid crystal formulation [14], peptide-hyaluronate conjugate [15] micelle-like nanoparticles [16,17], and liposomal compositions [18] have been reported. Although these methods exhibit certain advantages such as focused concentrated drug delivery to tumors, they also pose however serious drawbacks such as lack of standardization and reproducibility in the synthesis of macroscopic assemblies, consistency in drug loading and therefore precise drug dosing upon administration, longer half-life for clearance from blood, and longer retention in liver/kidneys causing side effects that are difficult to address. Further advancement of these potentially effective anticancer therapies is delayed because of their undesirable and intricate pharmacology. These difficulties can be circumvented by the conjugation of potentially promising anticancer agent with cancer targeting, biologically innocuous small molecules such as synthetic peptides [19], non-coding RNA [20] or heptamethine cyanine dyes [21]. Recently it has been demonstrated that a variety of cancer cell lines express higher levels of certain types of organic anion transport peptides (OATPs) and the hypoxic conditions in which tumors thrive possess special affinity towards certain selective heptamethine cyanine dyes [22,23]. Overexpression of OATPs allow enhanced transport of charged heptamethine cyanine dyes across the cell membrane and their storage in liposomes in cancer cells. In addition these dyes do not possess any apparent biological activities [22]. We recently demonstrated that radiolabeled cyanine dye molecules can be used to detect and diagnose cancer lesions [24,25]. Additional reports of cancer cell-specific delivery and targeting of drug-cyanine dye conjugates to tumors have been described by us and other laboratories [26,27,28,29,30,31,32]. Considering ongoing research on genistein in our laboratory [33], we sought to improve its therapeutic potential by conjugating it with a cyanine dye. Recent development along those lines are described here.

IR-783 is a well-defined heptamethine cyanine dye with high water solubility and a charged structure with two sulfonic acid side chains which make it a promising candidate carrier molecule to conjugate with genistein. IR-783 was shown to be recruited in cancer cells and did not exhibit any known cytotoxicity [34,35,36]. The chlorine atom on the bridging ring of IR-783 is known to be reactive towards a variety of nucleophiles, especially phenolate ion, [34,37]. Using this known chemistry, we synthesized the first genestein-IR-783 conjugate **4**. Following its detailed characterization and verification of high purity, an in vitro biological evaluation in the breast cancer cell line MCF-7 in comparison with noncancerous MCF-10A cells was first carried out. Cancer cell selective targeting ability, time and dose dependent accumulation in cells by confocal microscopy, quantitative estimation by LCMS analysis were demonstrated. A potential mechanism of action on uptake was investigated. The in vivo tumor targeting properties of genistein-IR783 conjugate **4** via near infrared fluorescence imaging were then acquired in MCF-7 breast cancer xenograft-bearing mice and the results acquired from these studies clearly demonstrated that conjugate **4** was accumulated in tumors.

## 2. Results

### 2.1. Chemistry

**Scheme 1:**
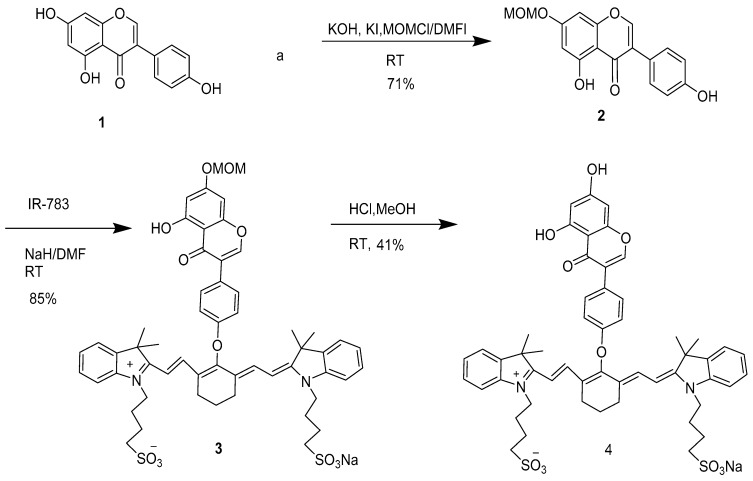
Detailed chemical synthesis scheme of the genistein-IR 783 conjugate **4**.

**Figure 1:**
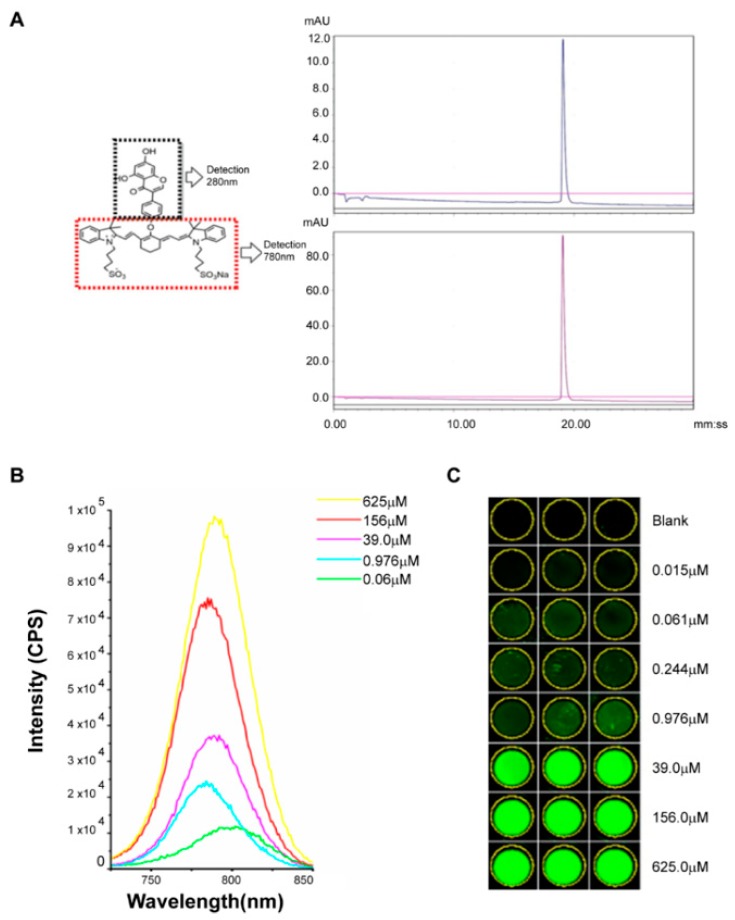
Characterization and purity of genistein-IR 783 conjugate **4**. Panel **A**. HPLC chromatographic characterization and purity assignment at two separate wavelengths indicating presence of both genistein (λmax = 280 nm) and IR783 (λmax = 750 nm) chromophores. Panel **B**. Fluorescence emission intensity measurements at varied concentration of conjugate **4** by fluorimeter (Excitation 750 nm). Panel **C**. Concentration dependent fluorescence intensity observation in 96 well/plate at 800 nm (emission) by Li-COR^®^.

Genistein was first selectively protected with a MOM group at the 7-OH position as previously reported [38], then the mono-MOM-protected genistein was subsequently deprotonated at the 4′-OH position with sodium hydride and the resulting 4′-phenolate was conjugated with IR 783. The crude product was treated with 1M HCl to remove the MOM protection. Purification of the conjugate on a silica column afforded a green solid in 86% overall yield as shown in Scheme 1. The structure of genistein-IR 783 conjugate was thoroughly characterized by spectroscopic analysis such as ^1^H-NMR and UV-VIS near-infrared fluorescence (NIRF) and MS data. Analytical HPLC was performed to ascertain the purity of genistein-IR783 conjugate **4** using a UV/VIS detector at two separate wavelengths (280 and 750 nm). The purity of the homogenous sample was observed to be >95% (Figure 1). All spectroscopic data were in agreement with the assignment of structure to the conjugate (see Supplementary Materials).

### 2.2. In Vitro Cell Uptake Assay

**Figure 2:**
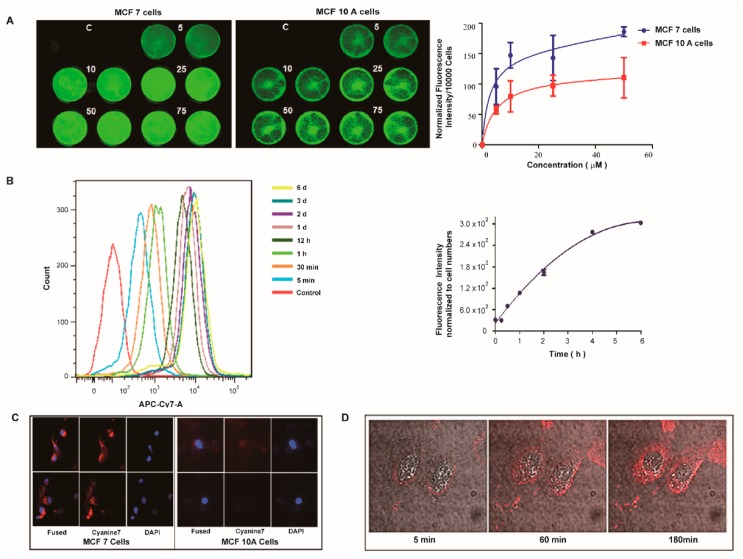
Concentration dependent comparative cellular uptake of Genistein-IR 783 conjugate **4**. Panel **A**. Intracellular uptake of conjugate **4** by MCF-7 and MCF-10A cells, as observed by fluorescence at various concentrations by Li-COR^®^. Panel **B**. Cell number normalized time dependent quantitative uptake as measured by fluorescence intensity by FACS cell analyzer, left panel observed histograms and right graphical depiction of rate of quantitative uptake. Panel **C**. Inverted fluorescence microscopic images of cell culture of MFC-7 and MCF-10 cells for confirmation of cell specific intracellular uptake of conjugate at 10× magnification. Panel **D**. Time dependent intracellular accumulation of conjugate in MCF-7 live cell as observed by continuous confocal microscopic imaging at 60× magnification.

Uptake of genistein-IR 783 conjugate **4** was evaluated in both in MCF-7 cells and MCF-10A cells. The qualitative and quantitative uptake of genistein-IR 783 conjugate was analyzed and measured by a variety of methods. First, by simply measuring the near-infrared fluorescence exhibited by cells upon incubation with conjugate **4** and normalizing with the protein content from cells in each well after thorough washing, although this is not true quantitative measure of conjugate uptake since the IR783 part of the molecule is emitting fluorescence, but it was a quick and semi-quantitative enough assay to compare the relative dose-dependent uptake. Figure 2 (Panel A) shows the concentration- dependent uptake in MCF-7 cells in comparison with MCF-10A cells and clearly indicates that uptake in MCF-7 cells was significantly higher than in MCF-10A cells at all concentrations tested. This observation is in agreement with the other related studies where heptamethine cyanine dyes were used to target cancer cells [22]. Fluorescence intensity per cell was analyzed under conditions of higher concentrations (5–75 M) and incubation for 4 h. Compared to MCF-10A, MCF-7 cells had more uptake in a dose-dependent manner when treated with genistein-IR 783 conjugate **4** (Figure 2, Panel A). In the second more sensitive experiment, fluorescence intensity measurements were performed on actual number of cells which consumed conjugate by cell counting (FACS) in a time dependent manner and quantified over time. Figure 2, Panel B left side depicts the fluorescence intensity histograms observed over time, which are graphically plotted in the right side for up to 6 h, to show rapid uptake. From FACS cell cytometry fluorescence measurements it appears that there were two distinct phases, first a rapid onset of uptake of conjugate **4** which happens over a 6 h period and later stabilizes over days to a slow and steady uptake. The cancer cell-selective cellular uptake and presence of genistein-IR 783 conjugate **4** was confirmed by inverted fluorescence and confocal microscopy of fixed cells from cell cultures. Fluorescence microscopic images of MCF-7 and MCF-10A cell cultures on cover slips (embedded in 6-well) under incubation with same concentration (10 M) genistein-IR 783 conjugate **4** for 14 h, were acquired. DAPI was used as nuclear stain for reference and Cy-7 channel (excitation at 750 nm, NIRF emission at 800 nm) was used for fluorescence. The images were processed with varying intensities of fluorescence signal for optimized image, the data as shown in Figure 2, panel C (see the Supplementary Materials for additional confocal imaging data on the uptake of conjugate **4** in MCF-7 and MCF-10A cells). The MCF-7 cells clearly show higher NIRF signals (Cy7, red color) as compared with MCF-10A cells, further confirming the cancer cell- specific targeting of genistein-IR 783 conjugate **4**. Figure 2, panel D depicts the live MCF-7 cell confocal imaging with time keeping all other conditions the same. An acquisition of fluorescence video over time convincingly confirms the intracellular accumulation of conjugate **4**.

### 2.3. Mechanism-Based Cellular Uptake of Conjugate

**Figure 3:**
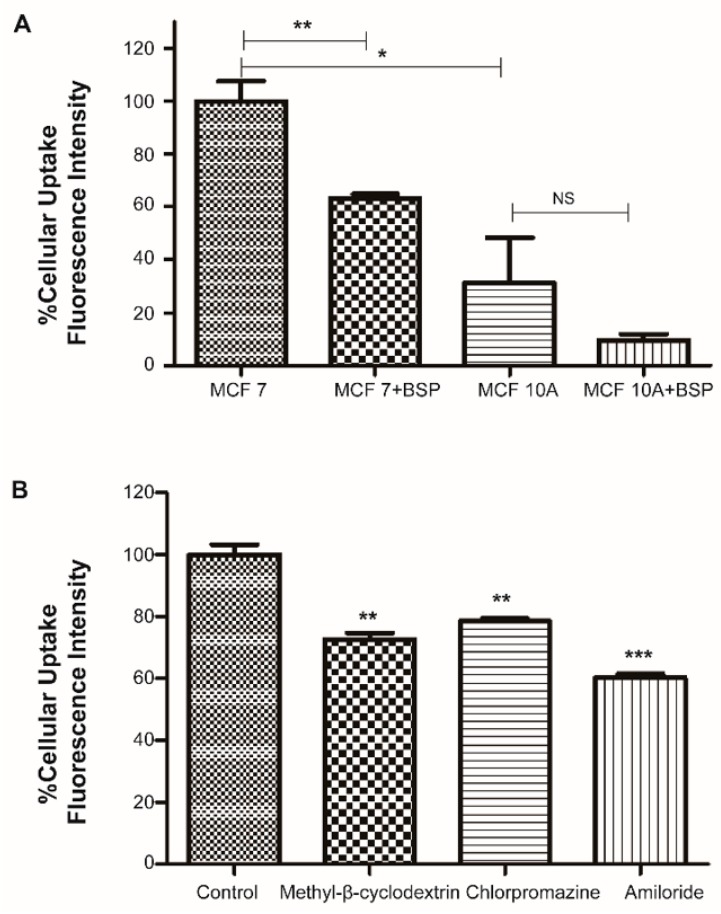
Mechanism based uptake of conjugate. Panel **A**. OATP inhibitor BSP inhibits conjugate uptake significantly in MCF-7 cells (*t*-test, two tailed *p* value 0.009), When compared with MCF-10A cells the uptake is not suppressed with BSP (*p* value 0.28). Comparison between MCF-7 and 10A cells is significant (*p* value 0.021). Panel **B**. As compared to the control group, chlorpromazine and, methyl-β-cyclodextrin and amiloride groups showed remarkable significance. Experiments were performed in triplicate and data represent as mean ± SD, significant difference versus control group, ∗ *p* < 0.05; ∗∗ *p* < 0.01; ∗∗∗ *p* < 0.001

MCF-7 and MCF-10A cells when incubated with genisteine-IR783 conjugate **4** in the presence and in the absence of OATP inhibitor BSP reveal the cancer cell selective inhibition of conjugate uptake in a significant way, as expected in MCF-7 cells (*t*-test, two tailed *p* value 0.009). The MCF-10A cells did not inhibit uptake in significant manner, (*t*-test two tailed *p* value 0.0928) as shown in Figure 3, panel A. In addition to OATPs, an endocytosis-based mechanism was also tested for uptake using three separate inhibitors. Clathrin-, caveolae- and macropinocyte-based endocytosis was evaluated by chlorpromazine, methyl-β-cyclodextrin and amiloride inhibitors, respectively, as reported previously [39,40]. It was observed that although all mechanisms played some role in the uptake, the micropinocytosis inhibitor had a significant impact on uptake, as shown in Figure 3, panel B.

### 2.4. In Vitro Cell Viability Studies.

**Figure 4:**
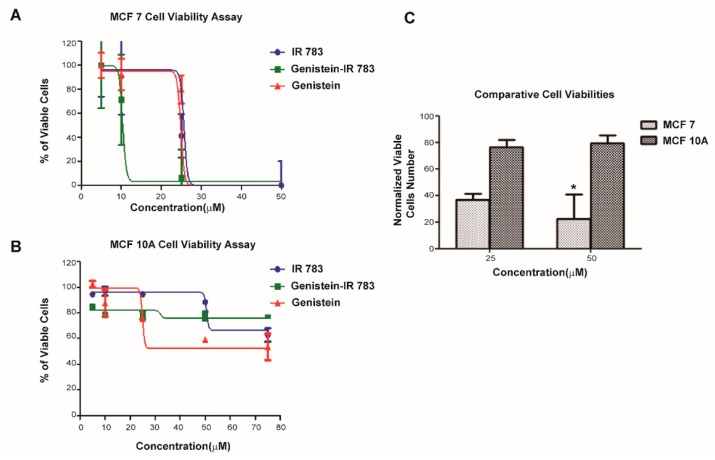
Determination of relative IC_50_ of genistein-IR 783 conjugate **4** on MCF-7 and MCF-10A cells. Viability of MCF-7 cells (panel **A**) and Viability of MCF-10A cells (panel **B**). Comparative cell viability at two separate concentrations of conjugate **4** in MCF-7 and MCF-10A cells (panel **C**).

The growth inhibitory effects of genistein-IR 783 conjugate **4** in comparison with genistein and IR 783 was next evaluated, first in MCF-7 cells at two separate concentrations (25 and 50 μM) to observe effect of conjugation. As shown in Figure 4 at both concentrations conjugate **4** was a more effective anticancer agent. Upon observing the relative effects of conjugate **4** as an anticancer agent, the cytotoxicity properties were evaluated by counting cell numbers of both MCF-7 and MCF-10A cells after a certain time of incubation. The conjugate **4** was observed to be more potent in MCF-7 cells than MCF-10A ones by the dose response curves. The dose response curves are presented in Figure 4 Panel B indicating improved potency of conjugate **4** (IC_50_ = 10.4 ± 1.0 μM) as compared with both parent genistein (IC_50_ = 24.8 ± 0.5 μM) and carrier IR783 (IC_50_ = 25.7 ± 0.7 μM). Especially carrier IR 783 is less toxic to normal cells (IC_50_ = 50.59 ± 0.3 μM) as compared with genistein (IC_50_ = 24.99 ± 0.3 μM). As shown in Figure 2, the uptake rate of genistein-IR 783 has reached the equilibrium after incubation with conjugate **4** for 3 days in MCF-7 cells, which indicated that concentration of conjugate **4** has reached the saturation level.

### 2.5. In Vivo Targeting Assay in Mouse Bearing Breast Tumor Xenograft.

**Figure 5:**
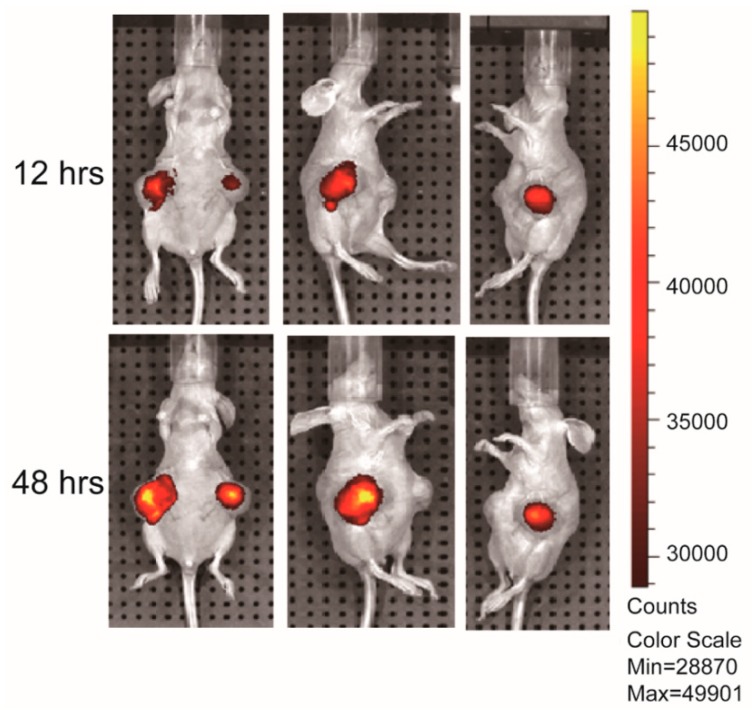
A representative example of an in vivo near infrared fluorescence imaging of mice bearing MCF-7 tumor xenografts injected with genistein-IR 783 conjugate **4** at two separate time points.

The tumor-targeting ability of genistein-IR 783 conjugate **4** in a MCF-7 breast cancer-bearing xenograft model was demonstrated by in vivo near infrared fluorescence imaging. Mice injected with genistein-IR 783 conjugate **4** (100 nM, intraperitoneally, i.p.) were live imaged upon establishing MCF-7 cancer xenograft in athymic ovariectomized mice as reported previously [41]. IVIS imaging post 12 h and 2 days injection of conjugate **4** clearly indicate the presence of near infrared fluorescence signal in tumors as shown in representative mouse in Figure 5.

## 3. Discussion

Genistein is an isoflavone natural product possessing a wide spectrum of biological activity, and its drug-like properties have been extensively studied to explore potential therapeutic effects. Especially the antineoplastic activity of genistein in various cancer cells such as breast, prostate, brain, colon and others has attracted huge interest [42,43]. Mechanistically the anticancer activity of genistein is attributed to variety of factors. It exhibits anti-angiogenic, protein-tyrosine kinase and DNA topoisomerase inhibitory and estrogen receptor modulatory activity amongst many other beneficial anticancer activities. All these anti-cancer activities ultimately result in disrupted intracellular signal transduction and/or DNA fragmentation at a molecular level thus resulting in suppressed cancer cell growth, induction of cell differentiation, apoptosis, and G2/M cell cycle arrest.

Genistein may cause these effects either separately or synergistically. However, to realize the optimal anticancer activity of genistein, it must be delivered to the tumor microenvironment and selectively taken up by cancer cells. Owing to genistein’s poor pharmacological properties such as low water solubility and moderate potency its full therapeutic potential is yet to be realized in in vivo studies. In order to expand the therapeutic potential of genistein as an anticancer agent via its delivery to the tumor environment and cancer cell selective uptake we sought to conjugate it with a heptamethine cyanine dye (HCD).

Certain heptamethine cyanine dyes are shown to possess special propensity to accumulate in cancer cells [21,32,44]. We proposed that upon conjugation with HCD, the bioavailability of genistein to the tumor would improve as the water solubility will be increased and cancer cell-selective uptake of the conjugate would further enhance the anti-cancer activity. Towards this goal a new genistein-HCD conjugate was successfully synthesized as follows: genistein possesses multiple phenolic hydroxyl groups (at the 4′, 5 and 7 positions), of which the 5-OH is the least reactive and is involved in hydrogen bonding with carbonyl groups. The 4′ and 7 phenolic hydroxyl groups of genistein were presumed to contribute poorly to its anti-cancer properties. The 4′-OH-glycosylated derivatives of genistein do not appear to be deleterious for anticancer activity [43,45]. First, it was difficult to control the reactivity between the 7 and 4′-OH groups resulting in the formation of regioisomers. In order to distinguish the reactivity between these two OH groups and to obtain a single conjugate, the protection of the 7-OH was first realized through a MOM group [38] since modification of the 4′-OH-hydroxyl group does not interfere with the observed biological activities. The 7-OH MOM-protected genistein was then selectively conjugated through the 4′-OH with IR 783. HCD, IR 783 is known to have high affinity with cancer cells and is now under extensive investigation from mechanistic to drug-carrying abilities [21,22,31,36,44]. As shown in Scheme 1 the desired conjugate was obtained as single isomer upon MOM group deprotection and characterized by ^1^H-NMR and mass spectrometry for the chemical composition (see Supplementary Materials). As expected, conjugate **4** did not display much change in near infrared fluorescence properties as compared with IR 783 (Figure 1).

Upon realization of genistein-IR 783 conjugate **4**, we next evaluated its cancer cell-targeting biological properties in MCF-7 breast cancer cells and compared it with that to equivalent noncancerous breast epithelial cells MCF-10A for selective targeting. The accumulation of conjugate **4** in MCF-7 cells was first analyzed by Li-COR as shown in Figure 2, panel A. The left side depicts well plate pictures, in which MCF-7 cells clearly have enhanced uptake of conjugate **4** in a concentration-dependent manner (from 5 μM to 75 μM) as compared with MCF-10A cells. The quantitative estimation of normalized fluorescence intensity in those two well plates are shown in right side of panel A. At all concentrations tested, the normalized fluorescence intensity per 1000 cells in both MCF-7 and MCF-10A cell lines clearly revealed that MCF-7 cells have increased uptake. Next the uptake of conjugate **4** in these cells with time at 25 μM was analyzed using Li-COR, as the incubation time is extended the uptake appears to increase faster at earlier time points (0 min. up to 4 h) and taper off 4 h later, as depicted in Figure 2B. Confocal microscopy of either fixed cells or live cells further confirmed the uptake of conjugate **4** at a microscopic level. Live cell imaging evidently confirmed with time the fluorescence intensity increased from 5 min through 3 h (Figure 2D).

Once the uptake of conjugate **4** was observed and validated, next we investigated its intracellular mechanism of uptake, especially since it has been reported that overexpression of certain types of organic anion transport peptides (OATPs) in several types of cell lines [23] are responsible for having high propensity to carry the heptamethine cyanine dyes inside cells. The overexpression of OATPs in breast cancer is an emerging area of research. Studies by Wlcke et al. showed higher expression of OATPs family between MCF-7 cells and MCF-10A cells because of OATP2A1 and OATP4C1, and they found that MCF-7 cells may be more sensitive than MCF-10A cells for OATP-specific anticancer agents [46]. In our study, BSP an inhibitor of OATPs effectively competes with conjugate **4** and suppresses uptake of conjugate **4** and causes significant inhibition (Figure 3A). However, because BSP is being nonselective inhibitor of OATPs, from the current studies we were unable to distinguish which subtype of OAPTs is responsible for the uptake of conjugate **4**. As the MCF-7 cell line is known to express a set of certain types of OATPs, it’s worth studying if some selective OATP is responsible for the uptake of conjugate **4**, but these studies were beyond the scope of the present work. In addition, endocytosis-mediated pathways for uptake were studied and a macropinocytosis inhibitor, amiloride, was found to be significantly affect the conjugate uptake (Figure 3B). These mechanistic studies indicated that multiple intricate pathways for cellular uptake of conjugate **4** are feasible and may thus overall result in relatively higher uptake in cancer cells versus normal cells.

Next, we evaluated the potency of genistein-IR 783 conjugate **4** in cell viability studies. From the dose-dependent studies of MCF-7 and MCF-10A cells it was clear that conjugate **4** was selective in targeting cancer cells. It was discovered that not only the conjugate was more potent to MCF-7 cells (10.4 ± 1.0 μM) than genistein (24.8 ± 0.5 μM) but it was less potent to MCF-10A cells (32.1 ± 1.1 μM), suggesting the possibility of cancer cell specific delivery (Figure 4A,B). These observations indeed support the notion that IR-783 is responsible for carrying genistein into the intracellular compartment of cancer cells far better than in normal cells, and this observation was in agreement with the other related studies where heptamethine cyanine dyes were used to target cancer cells [22]. These results also suggest that it will be also worth evaluating the cancer cell targeting ability of this conjugate **4** in other cell types. Due to the moderate potency of genistein as an anticancer agent it must be administered in comparatively high doses [43,47]. With limited cancer targeting properties it may cause undesired side effects and therefore its advancement as an anticancer agent was hindered. Conjugation with the heptamethine cyanine dye IR-783 improved the cancer cell-targeting properties of genistein in MCF-7 cells compared with non-cancer MCF-10A cells used as a control.

The delivery of conjugate **4** to the tumor microenviroment was then studied by imaging. In order to confirm tumor-specific targeting, mice bearing MCF-7 xenografts were injected with genistein-IR 783 conjugate **4** and in vivo near infrared imaging with an IVIS scanner was performed. As is evident from the images shown in Figure 5 tumors were visualized upon systemic administration. Although for therapeutic evaluation much more work is needed, the work presented here indeed indicates that the genistein-IR 783 conjugate **4** has the potential to be efficacious drug as compared with the parent genistein. Since the conjugate **4** improved the targeting profile of the parent genistein, it is worthy of further investigation. Whether this enhanced anti-cancer activity reflects in vivo therapeutic efficacy of breast cancer in a mice model needs to be validated. In future work we will evaluate the in vivo anticancer activity along with genistein and access the efficacy and toxicity of the conjugate **4**.

## 4. Conclusions

A conjugate of genistein with IR 783 has been successfully synthesized and thoroughly characterized. The conjugate **4** was then evaluated for cancer cell-selective targeting in the MCF-7 breast cancer cell line in comparison with non-cancerous MCF-10A cells. The results from our studies suggest that IR 783 acts as a superior carrier for the delivery of genistein to cancer cells. The conjugate exhibited better anti-cancer properties compared with the parent genistein. The results of this study prove our hypothesis that cancer cell targeting profiles of potential anticancer agents can be significantly improved by conjugating it with IR 783.

## 5. Materials and Methods

### 5.1. General Chemisty

Krebs-Henseleit (KH) buffer pH 7.4 was prepared as with 118 mM NaCl, 23.8 mM NaHCO_3_, 4.83 mM KCl, 0.96 mM KH_2_PO_4_, 1.20 mM MgSO_4_, 12.5 mM HEPES, 5.0 mM glucose, and 1.53 mM CaCl_2_. Final covalently attached conjugate was primarily characterized by ^1^H-NMR and its molecular composition was assigned via mass analysis. The purity of conjugate was analyzed and confirmed by HPLC. ^1^H-NMR data were collected on 300 or 500 MHz spectrometers (Varian, Palo Alto, CA, USA) using standard parameters; chemical shifts are reported in ppm (δ) with reference to residual non-deuterium labeled solvent. Coupling constants are reported in Hz. A preparative HPLC system Varian Prostar system equipped with pumps, 210; column valve module, 500; fraction collector, 701 and a PDA detector was utilized to purify newly synthesized intermediates and final compound using either DENALI™ 238 DE C18 SPRING preparative column (120 Å, 250 × 25 mm) and/or an Alltech Apollo C18 semi-preparative column (5 µm, 250 × 10 mm) with a Phenomenex C18 Security Guard cartridge system (10 × 10 mm I.D.) columns (Grace Davison Discovery Sciences, CA, USA). Two separate mobile phases: Solvent A (0.1% TFA in water) and Solvent B (0.1% TFA in 80% aqueous acetonitrile) were used to elute HPLC. The gradient for mobile phase was varied as per the need of purification of specific compounds and fractions were monitored at dual wavelengths 280 and 750 nm. A flow rate of 12 mL/min and 3 mL/min was implemented for the preparative and semi-preparative column chromatography respectively. Analytical HPLC was performed using Alltech’s Econosphere C18 reverse phase column (3 µm, 4.6 mm ID × 150mm) at a flow rate of 0.5 or 1.0 mL/min with gradient of Solvents A and B as required and monitored at 750 nm. Mass spectrometric analysis of new compounds was performed by matrix-assisted laser desorption/ionization time-of-flight mass spectrophotometer (MALDI-TOF-MS) at the University of Virginia’s the W.M. Keck Biomedical Mass Spectrometry Laboratory using a Bruker Daltonics system (Santa Clara, CA, USA). UV-Vis spectra were acquired on UV-Vis spectrometer with a path length of 1 cm with spectral width 250–800 nm (Biomate 5, Thermo Spectronic, Rochester, NY, USA) and analyzed by VISIONlite software (Thermo Scientific, Waltham, MA, USA). Fluorescence emission spectra for conjugate were acquired on Horiba FluoroMax 4 spectrofluorometer (JOBIN YVON/HORIBA, Edison, NJ, USA) with various excitation wavelengths (slit width 5 nm and integration time of 0.2 s) for all measurements. The emission spectra were acquired from 700–900 nm. Spectral data were analyzed and plotted with Origin software (Origin Lab, Northampton, MA, USA). A Li-COR^®^ instrument (LICOR Biotechnologies, Lincoln, NE, USA) was used for quantitative near infrared fluorescence (NIRF) signal measurement of cellular uptake studies. Emission wavelength of 800 nm was used for acquiring the fluorescence data with standard parameters. All parameters were kept constant for acquiring fluorescence intensity for given specific study and wherever necessary the fluorescence data was normalized to protein. An IX81 inverted microscope (Olympus, Shinjuku City, Tokyo, Japan) equipped with six-position fluorescence filter turret was used for confocal imaging of MCF-7 and MCF-10A cells as well as fixated tumor tissue using Cyanine 7 near infrared channel. Slidebook software (Version 6.0, Intelligent Imaging Innovations Inc. Denver, CO, USA) was utilized for image collection and data processing. Flow cytometry experiments were performed on BD FACSCANTOTM III Cell analyzer (FACSCAN, Becton Dickinson, San Jose, CA, USA). In vivo near infrared fluorescence (NIRF) imaging of breast cancer xenograft bearing mice was performed on an IVIS Spectrum instrument Xenogen (Caliper Life Science, Hopkinton, MA, USA) with an excitation wavelength of 745 nm and observed emission wavelength at 820 nm. The NIRF in vivo imaging data were analyzed and presented with Living Image software (4.1).

### 5.2. Synthesis and Characterization of Genistein-IR783 Conjugate

Briefly, genistein was first selectively MOM-protected at the 7-OH position (see Supplemental Materials for detailed procedures), the MOM-protected genistein was conjugated with IR 783 under basic conditions followed by MOM deprotection using dil. HCl (1.0 M) yielding the final genistein-IR 783 conjugate as shown in Scheme 1. The genistein-IR 783 conjugate **4** was first purified by column chromatography over silica gel and thoroughly characterized by spectroscopic analyses including ^1^H-NMR, UV-VIS, near infrared fluorescence (NIRF) and MS data. Prior to biological studies the purity and homogeneity of conjugate **4** was ascertained by analytical HPLC.

### 5.3. Spectroscopic Evaluation of Conjugate (UV-VIS and Fluorescence).

#### 5.3.1. UV-VIS Spectra

A solution (10 µM) of conjugate **4** was prepared in DMSO and serially diluted. UV-Vis absorption measurements of diluted solutions were acquired (250–800 nm) and the data were processed and analyzed with VISIONlite software.

#### 5.3.2. Fluorescence Spectra

Fluorescence emission spectra were acquired with spectral range of 400–900 nm, with 5 nm slit width, 0.2 s integration time for all measurements. Different concentrations for genistein-IR 783 conjugate **4** were prepared by dissolving the solid green sample in DMSO. Fluorescence spectra were measured by adding 3 mL aliquots of the stock solutions into a quartz cuvette with 1 cm path length. Spectra data were recorded and processed, fluorescence intensities were normalized and plotted with Origin software. The concentration dependent (0.060, 0.976, 39, 156 and 625 µM) fluorescence intensities were recorded for the genistein-IR 783 conjugate.

#### 5.3.3. Partition Coefficient

The hydrophilic characteristics of conjugate **4** was measured by dissolving it first in water and then adding octanol followed by vigorous mixing. In brief, the purified conjugate **4** (2.78 mg) in 0.5 mL water was mixed with 0.5 mL of *n*-octanol, and the resulting biphasic mixture was stirred thoroughly for 10 min, and centrifuged at 4000 rpm for 5 min. at final concentration of 2.83 µM (this concentration of solution was within the linear range to observe the correlation between fluorescence probe concentration and fluorescence intensity). Aliquots (in triplicate) from each organic and aqueous layer (50 µL) were carefully withdrawn with pipette and transferred into 96-well plate. The fluorescence intensity of individual well from the well plate was measured via the CCD camera of a Xenogen IVIS Spectrum system equipped with a filter set of λ_Ex/Em_ = 745/820 nm (auto exposure, medium bining, F stop = 2). Circular regions of interest (ROI) on each well were drawn for measuring fluorescence intensity of individual well in the form of average radiance (p/s/cm^2^/sr) using software Living Image 4.1. Log P was calculated using the equation: log ([fluorescence intensity in octanol]/[fluorescence intensity in water]) to access partition coefficient. Solvent corrected normalized fluorescence intensities were first used to calculate log P [48].

### 5.4. Cellular Uptake Assays

Uptake of genistein-IR 783 conjugate **4** by MCF-7 cells were evaluated by multiple methods for qualitative and quantitative analysis. IMEM containing 5% fetal bovine serum (FBS) (Invitrogen, Carlsbad, CA, USA) and 1% penicillin/streptomycin (GIBCO, CA, USA,) growth media was used for MCF-7 cell culture as described earlier [41]. Immortalized MCF-10A cells were derived from non-transformed epithelial cell line of human fibrocystic mammary tissue and cell culture was purchased from American Type Culture Collection (ATCC, VA, USA). Cell culture medium DMEM/F12 supplemented with epidermal growth factor 40 ng/mL (BioVision, Milpitas, CA, USA), cholera toxin 100 ng/mL (Calbiochem, Darmstadt, Germany), 5% horse serum (Invitrogen, Carlsbad, CA, USA), insulin 10 µg/mL (Sigma, St. Louis, MS, USA), hydrocortisone 500 ng/mL (Sigma), 1% penicillin and streptomycin were used to proliferate MCF-10A cells. All cell lines were cultured in the incubator at 37 °C in a 5% CO_2_ humidified atmosphere (CO_2_ water Jacketed incubator, series II, Forma Scientific, Merietta, OH, USA). MCF-7 and MCF-10A cells were cultured in their respective media as mentioned and were used for flowing studies [49]. In the first experiment, the comparative cell uptake was performed by measuring relative fluorescence intensity (emission 800 nm) of both MCF-7 and MCF-10A cells at various concentrations. Both cell types were plated in separate multiple six well plates and incubated for two days to attain about 80–90% confluence, the media from each well was removed and fresh media added along with genistein-IR 783 conjugate at various concentrations in triplicate for 4 h. For concentration-dependent studies the same plates were replicated for various concentrations of genistein-IR 783 conjugate incubation. The individual well NIRF signal intensity measurement in whole 96 well plate was performed on a Li-COR^®^ system (emission observed at 800 nm). The signal intensity from each well was normalized to protein content of the same well as measured by a standard Bradford assay (Bio-Rad, Hercules, CA, USA). The data was analyzed and presented with Prism Graphpad software version 5 (GraphPad Software Inc., La Jolla, CA, USA). In the second experiment, time-dependent cellular uptake measurements by FACS cell analyzer of MCF-7. The MCF-7 cells were plated in 6 well/plate (1 × 10^6^ cells/well) and incubated for two days to attain about 80–90% confluence, the medium was replaced with a fresh medium supplanted with genistein-IR 783 conjugate (1 μg/mL). The cells were further incubated for various time points at 37 °C in a 5% CO_2_ humidified atmosphere. At each time points cells in well were washed three times (1 mL) with PBS to remove the old medium and conjugated adhered to the cell surface. Finally cells were harvested, centrifuged at 1000 rpm for 3 min supernatant were removed, and cell pellet resuspended in PBS prior to cell analyzer experiment. The fluorescence intensity of cells from each well from whole plate was analyzed using a BD FACSCANTOTM III instrument.

### 5.5. Mechanistic Cellular Uptake Assays

#### 5.5.1. OATPs Inhibition 

MCF-7 and MCF-10A cells (~6 × 10^4^ per well) were seeded on 6-well culture plates and incubated at 37 °C in a 5% CO_2_ humidified atmosphere for 24 h. In order to determine whether genistein uptake and accumulation in cancer cells were dependent on OATPs, cells were divided to two groups: Group 1 was pre-treated with 250 μM bromosulfophthalein (BSP, a known competitive inhibitor of OATPs) for 1 h prior to incubating with the genistein-IR 783 conjugate **4** (25 µM); Group 2 was without BSP and only incubated with genistein-IR 783 (25 µM). After 4 h incubation, cells in both groups were washed with PBS containing 5% FBS. Li-COR was used to measure NIRF signal intensity of each well from whole plate and the protein content from each well was used to normalize NIRF intensity data as described above by a Bradford assay (Bio-Rad). Upon completion of treatment, cells from each well were washed twice with saline. Cells were counted using a Coulter counter for actual cell numbers by preparing as nuclei as follows: sequential addition of 1 mL HEPES-MgCl_2_ solution (0.01 M HEPES and 1.5 mM MgCl_2_) and 0.1 mL ZAP solution (0.13 M ethylhexadecyldimethylammonium bromide in 3% glacial acetic acid (*v*/*v*), Sigma), to suspended cells for fixing up nuclei for counting. GraphPad Prism software (was used for processing the cell count data.

#### 5.5.2. Macropinocytosis Inhibition 

About MCF-7 cells (1 × 10^6^ cells/well) were incubated with culture media in six well plates at 37 °C, after 24 h, the cells were preincubated with endocytic inhibitors including chlorpromazine (10 mg/mL), methyl-β-cyclodextrin (10 mmol/L) and amiloride (50 mmol/L) for 30 min at 37 °C in a 5% CO_2_ humidified atmosphere. Then, genistein-IR783 conjugate **4** (1 μg/mL) was added to cells for another 4 h. The cells were washed three times with PBS to remove the old medium from the cell surface. The cells were then harvested, centrifuged at 1000 rpm for 3 min, and resuspended in PBS. Finally, the cells were processed and examined using a BD FACS Canto III system (FACSCAN, Becton Dickinson, San Jose, CA, USA).

### 5.6. In Vitro Cell Viability Assays

About 6 × 10^4^ MCF-7 and MCF-10A cells were first individually incubated with culture media in six well plates at 37 °C, two days after incubation the media was removed and along with fresh media cells were independently treated with genistein, IR 783 and genistein-IR 783 conjugate **4** for 3 days in triplicate for separate well plates. Ethanol or DMSO was used to dissolve the conjugate and genistein and its final concentration was less than 0.1% in cell media during the experiment. Cells were washed carefully twice with saline after the treatment. For total cell counting nuclei were prepared by sequential addition of 1 mL HEPES-MgCl_2_ solution (0.01 M HEPES and 1.5 mM MgCl_2_) and 0.1 mL ZAP solution (0.13 M ethylhexadecyldimethylammonium bromide in 3% glacial acetic acid (*v*/*v*), Sigma), as standard protocol. Cells as fixed nuclei were finally counted on a Coulter counter for assessment of cell growth and viability [41,50,51].

### 5.7. In Vivo MCF-7 Breast Cancer Xenograft Bearing Mice Model

A breast tumor MCF-7 xenograft model was used for in vivo tumor targeting properties of genistein-IR 783 conjugate **4**. Protocol for Animal experiments (SCXK 2011-0029) was approved by the Guangzhou University of Chinese Medicine Animal Care and Use Committee (Guangzhou, China), and studies were conducted in accordance with the ethical standards and national guidelines. Female Balb/c nu/nu nude mice (4–6 weeks) were obtained from Laboratory Animal Center of Sun Yat-Sen University (Guangzhou, China). In addition, part of animal work performed at University of Virginia was approved by Animal Study Committee’s requirements for the care and use of laboratory animals in research, the mice were handled and processed according to current guidelines of National Institutes of Health Model Procedure of Animal Care and Use. Female athymic mice (3–4 weeks old) were obtained from Charles River Laboratory (Wilmington, MA, USA). All the mice were housed in a pathogen-free environment under controlled conditions of light and humidity and received food and water ad libitum for the course of studies.

### 5.8. In Vivo NIRF Imaging of Mice Bearing MCF-7 Breast Tumors

In an assigned group of mice (n = 5), 100nM of genistein-IR 783 conjugate **4** was injected intraperitoneally. Post 12 h and 48 h injection of conjugate NIRF images were acquired on Xenogen IVIS Spectrum instrument. Parameters for image acquisition were as follows: Excitation wavelength 745 nm, emission observed at 820 nm, with auto exposure (f/stop = 2). Imaging data were analyzed and presented at the same scale of fluorescence intensity with Living Image 4.1 software. Region of Interests (ROIs) measurement were obtained from all tumors by manual drawing of circles as available in software. The non-tumor bearing part of body of mice was used to measure as background ROIs. Subtracting the background ROIs from the tumor ROIs measurement revealed normalized fluorescence intensities for tumors. The fluorescence emission was reported as photons per second per centimeter square per steradian (p/s/cm^2^/sr) [52].

### 5.9. Statistical Analysis

All data were shown as means ± standard deviation. Student’s *t*-test is used from the GraphPad software to determine statistical significance. A *p* value of less than 0.05 was considered statistically significant. 

## Supplementary Materials

The following are available online at https://www.mdpi.com/1420-3049/24/22/4120/s1, Figure S1–S5: Spectra of compound **2**,**3** and **4**.
