# Supplementary material for: Synthesis and Biological Evaluation of Genistein-IR783 Conjugate: Cancer Cell Targeted Delivery in MCF-7 for Superior Anti-Cancer Therapy
Source: Molecules. 2019 Nov 14;24(22):4120. doi: 10.3390/molecules24224120 (PMC6891397; doi:10.3390/molecules24224120)
Supplement: Supplementary file 1 [file molecules-24-04120-s001.pdf]

Supplementary Materials

Synthesis and Targeted Evaluation of Genistein-IR783 Conjugate: Cancer Cell Targeted Delivery for Superior Anti-Cancer Therapy

Yang Guan[1, 2,3,#], Yi Zhang [3], Juan Zou [2], Li-Ping Huang [1,#], Mahendra D. Chordia [3], Wei Yue [4], Jin-Jun Wu [2,*] and Dongfeng Pan [3,*]

[1] Jiangxi University of Traditional Chinese Medicine, Nanchang,Jiangxi, 330004, China; guanyang1671@163.com (Y.G.), jxnchlp@163.com(L.-P.H.)

[2] International Institute for Translational Chinese Medicine, Guangzhou University of Chinese Medicine, Guangzhou, Guangdong 510006, China; zoujuangzy@163.com(J.Z.)

[3] Department of Radiology and Medical Imaging, Charlottesville, Virginia 22903, USA; zhangy87@gmail.com (Y.Z.); mdc3x@virginia.edu (M.D.C.)

[4] Department of Endocrinology, University of Virginia, Charlottesville, Virginia 22903, USA;; WY9C@hscmail.mcc.virginia.edu

[#] Authors equally contributed to the paper.

[*] Correspondence: DP3R@hscmail.mcc.virginia.edu (D.-F.P.); wujinjun@gzucm.edu.cn(J.-J.W.); Tel: +1 (434) 243-2893(D.-F.P.); +86-159-1435-8366(J.-J.W.)

General Methods

All chemicals commercially procured were from standard sources such as either Sigma-Aldrich (St. Louis, MO, USA) or Acros Organics (NJ, USA) and were used as is without further purification. Ultrapure water (resistivity, 18.2 MΩ cm) used for making solutions was obtained from Milli-Q Direct Ultrapure Water System from Millipore (Billerica, MA, USA). High-performance liquid chromatography (HPLC) grade solvents were purchased from Fisher Scientific (Pittsburgh, PA, USA). Intermediates and final compounds were purified by preparative and semi-preparative reversed-phase HPLC. Final product genistein-IR 783 conjugate 4 was thoroughly analyzed for purity by analytical HPLC using Alltech's Apollo C18 5 μ column. HPLC was performed on a Varian ProStar system (models: pumps, 210; column valve module, 500; fraction collector, 701) (Varian Instruments) equipped with either a DENALI™ 238 DE C18 SPRING preparative column (120 Å, 250 × 25 mm) and an Alltech Apollo C18 semi-preparative column (5 μm, 250 × 10 mm) (Grace Davison Discovery Sciences) using PDA detector. Two mobile phases were used for HPLC: Solvent A (0.1% TFA in water) and Solvent B (0.1% TFA in 80% aqueous acetonitrile). The mobile phase gradient varied according to characteristics of compounds for 30 min at a flow rate 12 mL/min for the preparative column and 3 mL/min for the semi-preparative column monitored at 750 nm. ^{1}H and ^{13}C-NMR data for intermediates (especially for compound **2–3**) were collected on Varian Unity 300 MHz spectrometer using standard parameters, and chemical shifts are reported in ppm unit with reference to residual non-deuterated solvent. MALDI-TOF mass spectra for compounds were obtained on a Bruker Daltonics system at W.M. Keck Biomedical Mass Spectrometry Laboratory of the University of Virginia.

Spectroscopic Evaluation

UV-Vis spectra: Solutions (0.1–10 μM) of IR-784 and Gensistein-IR 783 conjugate **4** were prepared separately in 0.1 M PBS buffer (pH 7.45) with 0.25% (*v*/*v*) DMSO. Measurement of absorption spectra were performed on UV-Vis spectrometer with the path length of 1 cm (Biomate 5, Thermo Spectronic, Rochester, NY, USA), scanned from 250-800 nm and analyzed by VISIONlite software. Molar extinction coefficients (ε) were then calculated.

Fluorescence spectra: Fluorescence emission spectra were performed with a Horiba FluoroMax 4 spectrofluorometer (JOBIN YVON/HORIBA, Edison, NJ, USA) with a variety of excitation wavelengths, with slit width of 5 nm and integration time of 0.2 s for all measurements. Spectra data were recorded, processed and plotted with Origin software. A solution of conjugate **4** (10 μM, 3 mL) was prepared in 0.1 N PBS buffer (pH 7.45) with 0.25% (*v*/*v*) DMSO and was excited with a series of excitation wavelengths (650, 675, 700, 725, 750 and 775 nm) to optimize the fluorescence emission spectrum. All spectra were normalized to the maximum emission intensity and plotted against emission wavelength.

Concentration-dependent fluorescence emission: Stock solutions (100 μM, 10 mL) of IR 783 and conjugate **4** were freshly prepared in 0.1 M PBS buffer (pH 7.45) with 0.25% (*v*/*v*) DMSO and a series of dilutions were made with PBS. Fluorescence emission spectra of both compounds were measured over the concentration range of 0–100 μM with the excitation wavelength of 750 nm while keeping rest of conditions the same as above. Maximum fluorescence intensities were plotted against each concentration.

Synthesis of Compound 2:

Genistein (**1,** 5.0 g, 18.5 mmol) was dissolved in *N*,*N*-dimethylformamide (40 mL), and to this mixture KOH (1.1 g, 18.5 mmol) was added, followed by potassium iodide (0.3 g, 1.85 mmol). The mixture was stirred at room temperature for 1 h and then to it chloromethyl methyl ether (1.68 mL, 22.2 mmol) was added dropwise with an addition funnel. The mixture was further stirred at room temperature for 12 h. The reaction mixture was poured into deionized water (120 mL), and the pH of solution was adjusted to 5.0 using 10% HCl, white solid precipitated, was collected by filtration and dried. Upon recrystallization from ethanol yielded 3.6 g, 61.5% product. M.P. 151~154. ^{1}H-NMR (400 MHz, DMSO-d_6) δ ppm: 3.42 (s, 3H, OCH_3), 5.21 (s, 2H, OCH_2O), 6.38 (d, *J* = 2.0 Hz, 1H, 6-H), 6.57 (d, *J* = 2.0 Hz, 1H, 8-H), 6.87 (d, *J* = 8.0 Hz, 2H, 3',5'-H), 7.47 (d, *J* = 8.0 Hz, 2H, 2',6'-H), 8.32 (s, 1H, 2-H), 12.93 (s, 1H, 5-OH). Mass [*m*/*z* 315 (M+)] observed for $C_{17}H_{14}O_6$.

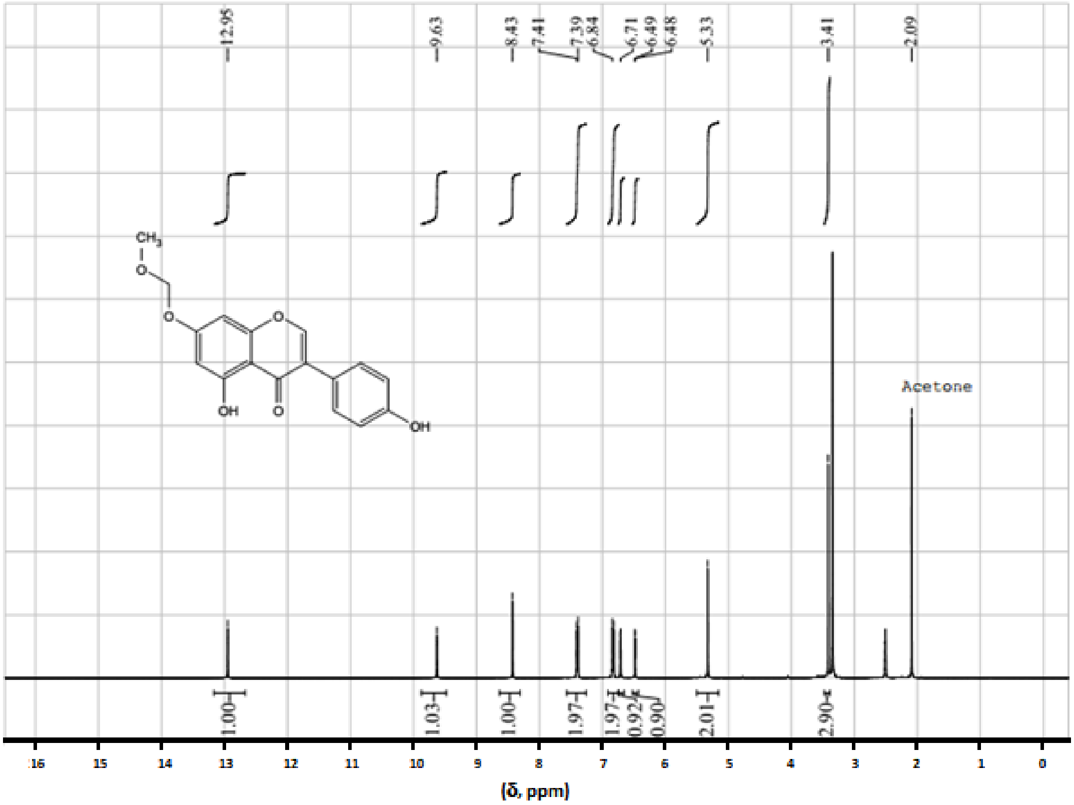

Figure S1. ^{1}H-NMR of 7-OH MOM protected genistein.

Synthesis of genistein-7-O-MOM-IR783 conjugate 3

7-O-MOM-genistein (462.0 mg, 1.47 mmol) was dissolved in dry DMF cooled down to 0 °C and NaH (230.0 mg, 5.75 mmol, 60%) was added to it and stirred for 10 minutes. A solution of IR-783 (1.11 g, 1.48 mmol) in 1:1 DMF:MeOH (40 mL) was added to above mixture and the dark green colored solution stirred overnight for 18 h. Methanol and volatiles were removed under reduced pressure and the solution was added to 50 mL diethyl ether and stirred vigorously and allowed to stand for 5 minutes, the supernatants were removed and the left over oily residue was treated two more times with 25 mL of diethyl ether to obtain a dark solid, which was dried and treated with water (10 mL). The solid crashed out was collected by filtration, dried (1.28 g, 85%). The solid was used as is for the next reaction. The conjugate **3** was characterized by NMR and mass spectroscopy as follows: ^{1}H-NMR (500 MHz, CD_3OD) δ ppm: 1.38 (s, 12 H, CH_3 × 4), 1.75–2.11 (m, 10 H), 2.79 (t, *J* = 5.0 Hz, 4H, CH_2), 2.88 (t, *J* = 5.0 Hz, 4H, CH_2), 3.48 (s, 3H, OCH_3), 4.15 (t, *J* = 5.0 Hz, 4H, CH_2 × 2), 5.27 (s, 2H, CH_2), 6.22 (d, *J* = 15.0 Hz, 2H, ArH), 6.45 (s, 1H, CH), 6.63 (s, 1H, CH), 7.20 (m, 4H, ArH), 7.30 (d, *J* = 10.0 Hz, 2H, ArH), 7.37 (m, 4H, ArH) 7.64 (d, *J* = 10.0 Hz, 2H, ArH), 8.01 (d, *J* = 15.0 Hz, 2H, =CH), 8.18 (s, 1H, ArH) 8.38 (s,1H, exchangeable OH). MS [*m/z* 1026 (M+)] for $C_{55}H_{59}N_2NaO_{12}S_2$.

Deprotection of 7-O-MOM of Genistein-IR conjugate 3: Synthesis of compound 4.

To a solution of compound **3** (1.03 mg, 1.0 mmol) in methanol (40 mL) was added 1.0 M HCl (10.0 mL) at room temperature and stirred for 4 h. Methanol was removed under reduced pressure and water was added to the residue, whereupon a solid crashed out that was collected by filtration. The solid was dried using a freeze dryer and purified using silica gel column chromatography using an acetonitrile/acetonitrile:methanol (1:1) gradient. Fractions indicating a single homogenous spot on TLC were collected and concentrated under reduced pressure to yield a dark green solid (393 mg, 41%). Conjugate **4** was characterized by ^{1}H-NMR, mass spectral analysis and fluorescence spectrometry. ^{1}H-NMR (500 MHz, DMSO-d_6) δ ppm: 1.29 (s, 12 H, CH_3 × 4), 1.75–2.22 (m, 10H), 1.94 (m, 4H, CH_2), 2.74 (t, *J* = 5.0 Hz, 4H, CH_2), 4.13 (t, *J* = 5.0 Hz, 4H, CH_2 × 2), 6.22 (d, *J* = 15.0 Hz, 2H, ArH), 6.38 (s, 1H, CH), 6.63 (s, 1H, CH), 7.18 (m, 4H, ArH), 7.24 (d, *J* = 10.0 Hz, 2H, ArH), 7.35 (m, 4H, ArH) 7.48 (d, *J* = 10.0 Hz, 2H, ArH), 7.81 (d, *J* = 10.0 Hz, 2H, ArH), 7.83 (brd, *J* = 10.0 Hz, 2H, =CH), 8.18 (s, 1H, ArH) 8.36 (s,1H, exchangeable OH). MS [*m/z* 959 (M-)] for: $C_{53}H_{56}N_2O_{11}S_2$ and high resolution mass calculated for $C_{53}H_{55}N_2O_{11}S_2Na$, 982.3145 and observed [*m/z* 982.2986 (M-H)-]. The conjugate was further purified for biological studies by preparative reverse phase HPLC and checked for purity at two separate wavelengths.

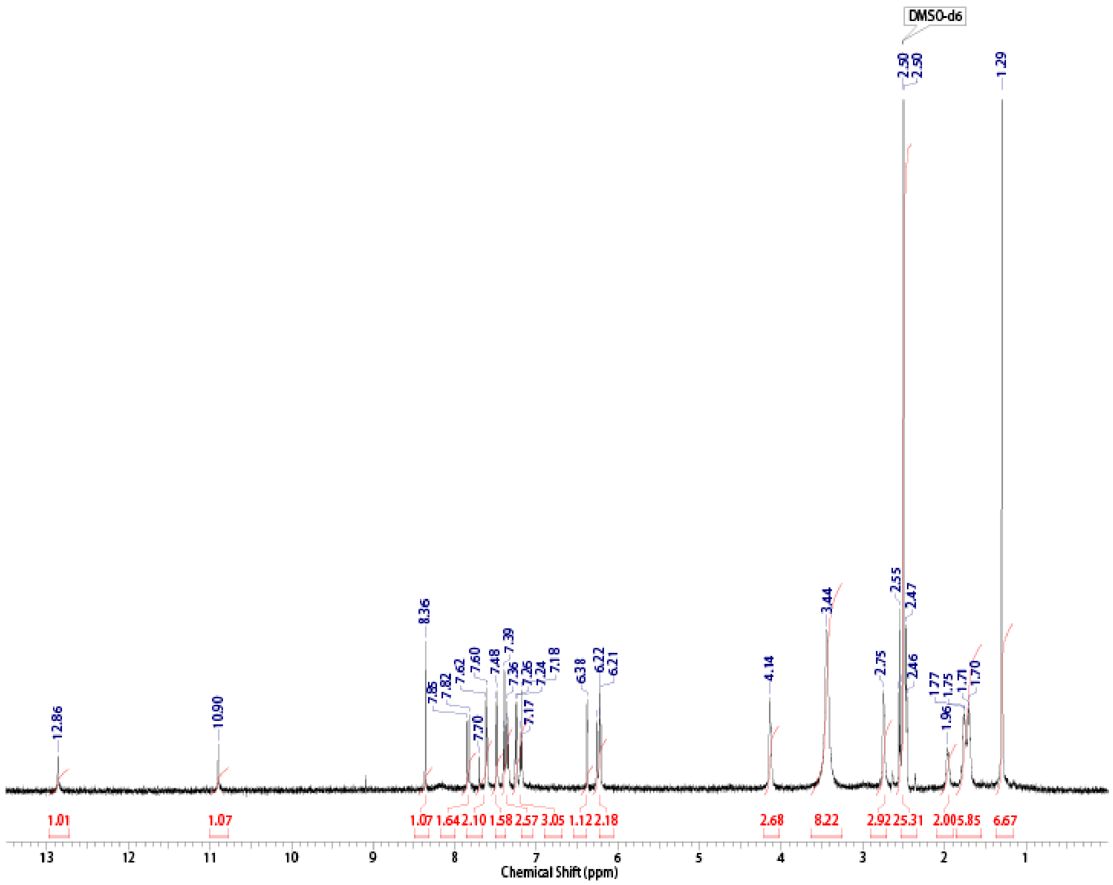

Figure S2. ^{1}H-NMR of genistein-IR 783 conjugate **4.**

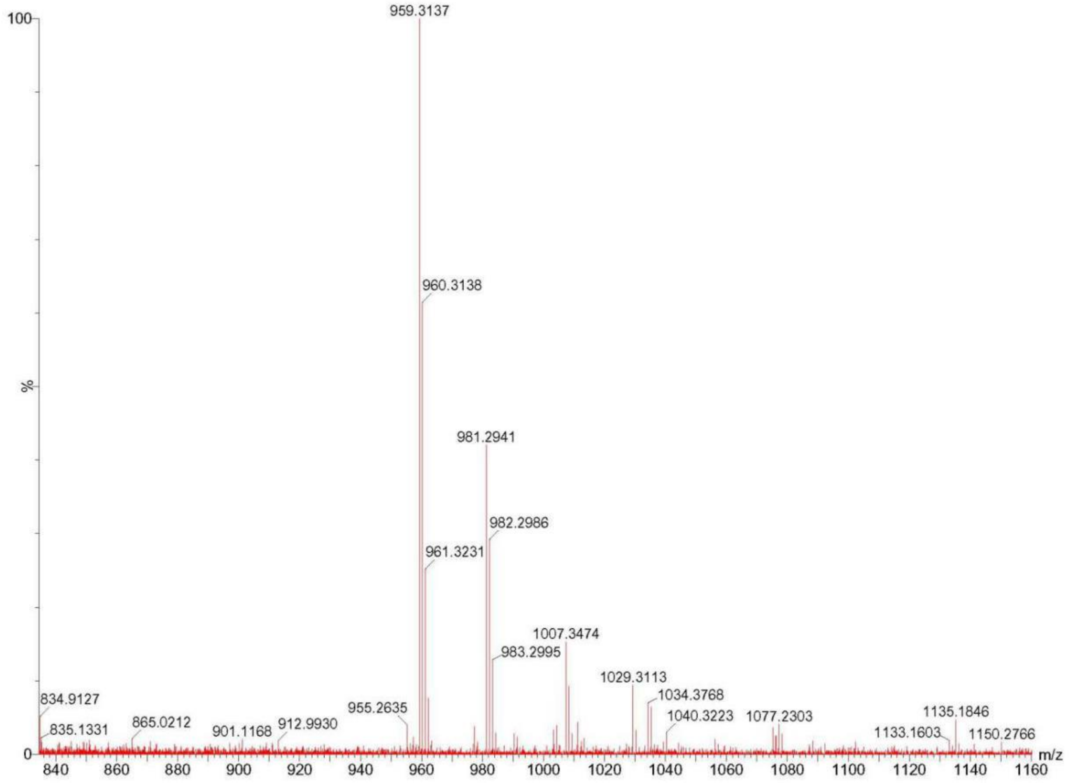

Figure S3. High resolution mass spectrum of genistein-IR 783 conjugate **4.**

A

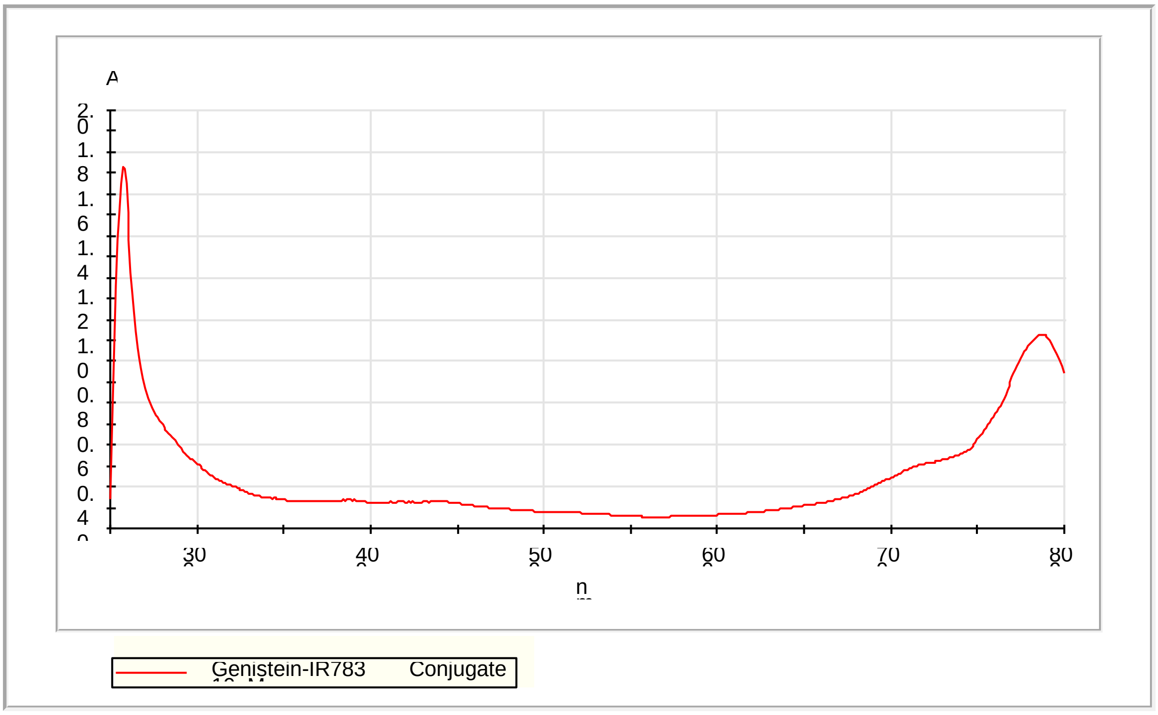

B

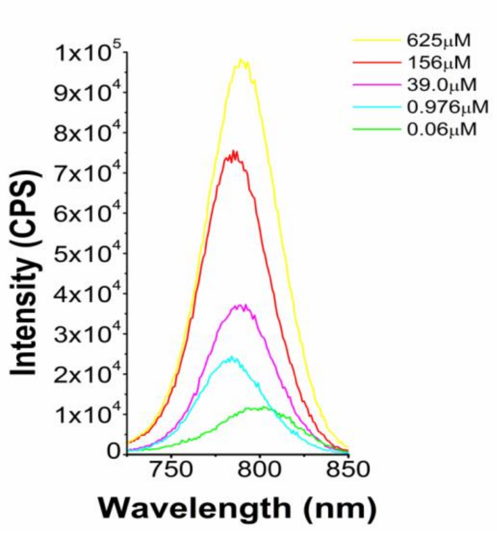

Figure S4. A: UV-Vis Spectrum of genistein-IR 783 conjugate 4 at 10 μM concentration. **B**: Spectrum of genistein-IR 783 conjugate 4 at different concentration.

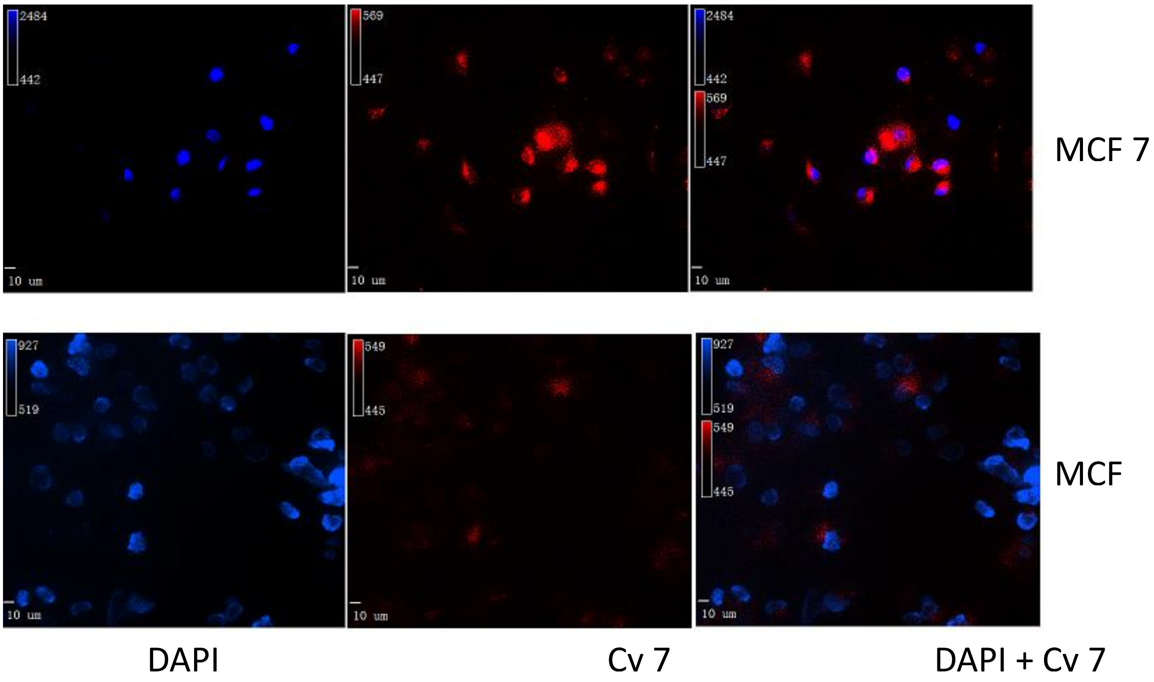

Figure S5. Panel C. Inverted confocal fluorescence microscopic images of MCF 7 and MCF-10A cell cultures on cover slips at 10× magnification.
